# Genome Sequence of the Sulfate-Reducing Bacterium *Desulfotomaculum hydrothermale* Lam5^T^

**DOI:** 10.1128/genomeA.00114-12

**Published:** 2013-01-24

**Authors:** Oulfat Amin, Marie-Laure Fardeau, Odile Valette, Agnès Hirschler-Réa, Valérie Barbe, Claudine Médigue, Benoit Vacherie, Bernard Ollivier, Philippe N. Bertin, Alain Dolla

**Affiliations:** a Aix-Marseille Université, Université Sud Toulon, CNRS/INSU, IRD, MIO-UM110, Marseille, France; b Aix-Marseille Université, CNRS, LCB-UMR7283, Marseille, France; c Laboratoire de Finition CEA, Institut de Génomique-Génoscope, Évry, France; d Laboratoire d’Analyses Bioinformatiques pour la Génomique et le Métabolisme (LABGeM), Genoscope-IG-CEA, Évry, France; e CNRS, UMR7156, Université Louis Pasteur, Strasbourg, France

## Abstract

Here, we report the draft genome sequence of *Desulfotomaculum hydrothermale*, a sulfate-reducing, spore-forming bacterium isolated from a Tunisian hot spring. The genome is composed of 2.7 Mb, with a *G+C* content of 49.48%, and it contains 2,643 protein-coding sequences.

## GENOME ANNOUNCEMENT

Members of the genus *Desulfotomaculum* are sulfate-reducing, spore-forming Gram-positive bacteria that are widely distributed within mesothermic and thermal ecosystems (1). Evidence of their presence in terrestrial geothermal hot springs has been obtained several times using molecular approaches (2), but only three species of this genus have been isolated so far, the most recent being *Desulfotomaculum hydrothermale* (3). Here, we report the first draft genome sequence of a *Desulfotomaculum* species isolated from a terrestrial hot spring in Tunisia: *D. hydrothermale*. The strain was shown to reductively detoxify arsenate [As(V)], in the presence of pyruvate as an electron donor, without evidence for any respiration using As(V) as a terminal electron acceptor (3). Because arsenic is known to be present in hot springs (4), information regarding the genome sequence of *D. hydrothermale* may be useful in understanding the As-detoxifying process developed in *Desulfotomaculum* species.

A Sanger/pyrosequencing hybrid approach was used for the whole-genome sequencing. A shotgun library was constructed with 10-kb fragments of genomic DNA in vector pCNS (derived from pSU). Sequencing with vector-based primers was carried out using the ABI 3730 Applera sequencer, and a total of 6,421 reads (~1.6-fold coverage) were analyzed and assembled with Newbler (a Roche assembler), with 341,086 single reads (~26-fold coverage) and 106,386 mate-paired 8-kb reads obtained with Genome Sequencer version FLX and Titanium (Roche Applied Science), respectively. The finishing step was achieved by using primer walks, PCRs, and *in vitro* transposition technologies. For quality assessment, 5,501,511 (36-nucleotide) Illumina reads (~70-fold coverage) were mapped using Soap1 (http://soap.genomics.org.cn/) on the 16 consensus contigs. The draft genome sequence consists of 2,706,432 bases with a *G+C* content of 49.48%, and it contains 2,643 candidate protein-encoding genes, in addition to 60 tRNA and 3 16S rRNA genes.

The mechanism of microbe-mediated metal reduction, studied mainly in *Geobacter* and *Shewanella* species, involves *c*-type cytochromes (5, 6). For *Desulfotomaculum reducens*, it has been hypothesized that an integral membrane multiheme *c*-type cytochrome (Dred_0700-0701) is involved in the reduction of metals such as Fe(III) and U(VI) (7). Inspection of the draft genome sequence of *D. hydrothermale* suggests the absence of *c*-type cytochromes and the absence of an identified ortholog of Dred_0700-0701, even though the strain is able to reduce Fe(III) to Fe(II) (3). Metal reduction in this strain would use a different pathway, which needs to be specified. The *D. hydrothermale* genome encodes an arsenite resistance protein (DESHYv2_110039) that exhibits homology to the intrinsic membrane protein ArsB (8). A gene (DESHYv2_110038) encoding a transcriptional regulator belonging to the AsrR family (9) was found immediately upstream from *asrB*. Even if no homolog to the arsenate reductase gene *asrC* (10) has been identified so far, this gene cluster could be of great importance for arsenite reduction and for the tolerance capacity of this strain.

Insights into the arsenite resistance process of *Desulfotomaculum* species could be gained through comparative genomics and biochemical analyses of the arsenite resistance mechanisms, which are in progress in our laboratories.

### Nucleotide sequence accession numbers.

The whole-genome shotgun project has been deposited at DDBL/EMBL/Genbank under the accession no. CAOS01000001 to CAOS01000016.
